# Supplementary material for: Short-Chain Fatty Acids Promote Mycobacterium avium subsp. hominissuis Growth in Nutrient-Limited Environments and Influence Susceptibility to Antibiotics
Source: Pathogens. 2020 Aug 26;9(9):700. doi: 10.3390/pathogens9090700 (PMC7559849; doi:10.3390/pathogens9090700)
Supplement: Supplementary file 1 [file pathogens-09-00700-s001.zip › pathogens-901209supp.pdf]

Biolog PM1 plate						
**Wells**	**Compounds**	**Planktonic MAH ($O.D._{590ηm}$)**		**Biofilm MAH ($O.D._{590ηm}$)**		**Abiotic Reaction**
		Mean	SD[#]	Mean	SD[#]	
A1	negative control	0.13	0.012	0.07	0.002	0.095
A2	L-Arabinose	0.28	0.048	0.11	0.064	0.324
A3	N-Acetyl-DGlucosamine	0.13	0.001	0.09	0.027	0.104
A4	D-Saccharic Acid	0.15	0.002	0.09	0.043	0.116
A5	Succinic Acid	0.14	0.012	0.08	0.010	0.551
A6	D-Galactose	0.15	0.003	0.09	0.043	0.142
A7	L-Aspartic Acid	0.15	0.002	0.07	0.007	0.119
A8	L-Proline	0.16	0.020	0.08	0.014	0.11
A9	D-Alanine	0.14	0.015	0.12	0.097	0.104
A10	D-Trehalose	0.14	0.011	0.09	0.038	0.091
A11	D-Mannose	0.16	0.003	0.10	0.058	0.127
A12	Dulcitol	0.14	0.014	0.08	0.025	0.1
B1	D-Serine	0.15	0.013	0.10	0.031	0.11
B2	D-Sorbitol	0.14	0.009	0.09	0.041	0.104
B3	Glycerol	1.28*	0.160	0.28*	0.170	0.093
B4	L-Fucose	0.16	0.009	0.09	0.030	0.129
B5	D-Glucuronic Acid	0.16	0.017	0.12	0.051	0.124
B6	D-Gluconic Acid	0.15	0.005	0.08	0.028	0.115
B7	D,L-α-Glycerol Phosphate	0.14	0.016	0.08	0.017	0.092
B8	D-Xylose	0.36	0.048	0.12	0.065	0.403
B9	L-Lactic Acid	0.15	0.018	0.09	0.019	0.096
B10	Formic Acid	0.17	0.019	0.12	0.042	0.09
B11	D-Mannitol	0.15	0.016	0.11	0.047	0.092
B12	L-Glutamic Acid	0.15	0.011	0.12	0.078	0.108
C1	D-Glucose-6- Phosphate	0.14	0.006	0.09	0.048	0.088
C2	D-Galactonic Acid-γ-Lactone	0.14	0.001	0.08	0.020	0.102
C3	D,L-Malic Acid	0.14	0.003	0.08	0.019	0.089
C4	D-Ribose	0.52	0.056	0.14	0.080	0.521
C5	Tween 20	0.72*	0.030	0.13*	0.062	0.116
C6	L-Rhamnose	0.18	0.009	0.10	0.043	0.223
C7	D-Fructose	0.18	0.009	0.09	0.019	0.147
C8	Acetic Acid	0.37*	0.150	0.14*	0.069	0.104

C9	α-D-Glucose	0.18	0.012	0.09	0.027	0.119
C10	Maltose	0.15	0.007	0.09	0.040	0.084
C11	D-Melibiose	0.14	0.002	0.11	0.063	0.094
C12	Thymidine	0.14	0.018	0.13	0.101	0.104
D1	L-Asparagine	0.15	0.001	0.07	0.007	0.109
D2	D-Aspartic Acid	0.15	0.004	0.09	0.031	0.09
D3	D-Glucosaminic Acid	0.14	0.010	0.08	0.012	0.105
D4	1,2-Propanediol	0.13	0.013	0.08	0.023	0.085
D5	Tween 40	0.58*	0.036	0.15*	0.056	0.103
D6	α-Keto-Glutaric Acid	0.24*	0.036	0.09	0.019	0.104
D7	α-Keto-Butyric Acid	0.24*	0.045	0.12	0.026	0.108
D8	α-Methyl-DGalactoside	0.15	0.010	0.08	0.007	0.111
D9	α-D-Lactose	0.13	0.019	0.08	0.019	0.098
D10	Lactulose	0.14	0.007	0.08	0.029	0.095
D11	Sucrose	0.15	0.002	0.09	0.033	0.083
D12	Uridine	0.14	0.010	0.10	0.040	0.089
E1	L-Glutamine	0.13	0.011	0.13	0.122	0.094
E2	m-Tartaric Acid	0.16	0.012	0.11	0.081	0.122
E3	D-Glucose-1- Phosphate	0.15	0.006	0.11	0.078	0.088
E4	D-Fructose-6- Phosphate	0.20	0.011	0.09	0.028	0.271
E5	Tween 80	0.49*	0.052	0.14*	0.045	0.111
E6	α-Hydroxy Glutaric Acid-γ- Lactone	0.15	0.022	0.09	0.025	0.079
E7	α-Hydroxy Butyric Acid	0.23*	0.035	0.14	0.027	0.09
E8	β-Methyl-DGlucoside	0.14	0.018	0.10	0.024	0.092
E9	Adonitol	0.16	0.003	0.09	0.022	0.106
E10	Maltotriose	0.15	0.012	0.11	0.040	0.095
E11	2-Deoxy Adenosine	0.13	0.025	0.07	0.034	0.097
E12	Adenosine	0.14	0.016	0.09	0.041	0.123
F1	Glycyl-L-Aspartic Acid	0.15	0.018	0.10	0.034	0.09
F2	Citric Acid	0.12	0.007	0.08	0.020	0.09
F3	m-Inositol	0.14	0.016	0.08	0.012	0.107
F4	D-Threonine	0.15	0.031	0.09	0.006	0.107
F5	Fumaric Acid	0.14	0.036	0.11	0.054	0.087
F6	Bromo Succinic Acid	0.18	0.011	0.15	0.046	0.191
F7	Propionic Acid	0.88*	0.227	0.17*	0.004	0.088
F8	Mucic Acid	0.12	0.008	0.12	0.066	0.09

F9	Glycolic Acid	0.13	0.008	0.08	0.020	0.095
F10	Glyoxylic Acid	0.19	0.024	0.12	0.040	0.19
F11	D-Cellobiose	0.17	0.020	0.12	0.044	0.093
F12	Inosine	0.13	0.009	0.13	0.057	0.093
G1	Glycyl-LGlutamic Acid	0.14	0.006	0.12	0.057	0.093
G2	Tricarballylic Acid	0.15	0.004	0.12	0.051	0.084
G3	L-Serine	0.14	0.002	0.10	0.023	0.108
G4	L-Threonine	0.16	0.029	0.12	0.044	0.102
G5	L-Alanine	0.14	0.016	0.13	0.033	0.097
G6	L-Alanyl-Glycine	0.14	0.012	0.11	0.012	0.14
G7	Acetoacetic Acid	0.21*	0.054	0.13	0.009	0.114
G8	N-Acetyl-β-DMannosamine	0.15	0.018	0.09	0.011	0.098
G9	Mono Methyl Succinate	0.48*	0.269	0.21	0.131	0.087
G10	Methyl Pyruvate	0.29*	0.038	0.16*	0.044	0.091
G11	D-Malic Acid	0.13	0.005	0.11	0.022	0.086
G12	L-Malic Acid	0.14	0.012	0.11	0.024	0.106
H1	Glycyl-L-Proline	0.13	0.007	0.09	0.014	0.126
H2	p-Hydroxy Phenyl Acetic Acid	0.13	0.005	0.09	0.037	0.081
H3	m-Hydroxy Phenyl Acetic Acid	0.11	0.007	0.08	0.028	0.079
H4	Tyramine	0.16	0.017	0.07	0.010	0.099
H5	D-Psicose	0.15	0.008	0.10	0.023	0.131
H6	L-Lyxose	0.48	0.071	0.13	0.058	0.55
H7	Glucuronamide	0.17	0.009	0.10	0.027	0.137
H8	Pyruvic Acid	0.27*	0.059	0.13*	0.007	0.107
H9	L-Galactonic Acid-γ-Lactone	0.17	0.029	0.08	0.015	0.118
H10	D-Galacturonic Acid	0.16	0.014	0.10	0.027	0.114
H11	Phenylethylamine	0.11	0.005	0.10	0.044	0.096
H12	2-Aminoethanol	0.13	0.016	0.16	0.132	0.096

**Biolog PM2a plate**						
**Wells**	**Compounds**	**Planktonic MAH ($O.D._{590\eta m}$)**		**Biofilm MAH ($O.D._{590\eta m}$)**		**Abiotic Reaction**
		Mean	SD[#]	Mean	SD[#]	
A1	Negative control	0.21	0.078	0.07	0.004	0.09
A2	Chondroitin Sulfate C	0.21	0.061	0.12	0.093	0.11
A3	Alpha-Cyclodextrin	0.20	0.077	0.09	0.049	0.09

A4	Beta-cyclodextrin	0.20	0.076	0.09	0.049	0.12
A5	Gamma- Cyclodextrin	0.20	0.066	0.09	0.049	0.12
A6	Dextrin	0.25	0.096	0.10	0.057	0.13
A7	Gelatin	0.22	0.095	0.11	0.060	0.08
A8	Glycogen	0.20	0.089	0.11	0.067	0.12
A9	Inulin	0.31	0.133	0.11	0.060	0.14
A10	Laminarin	0.19	0.070	0.12	0.077	0.08
A11	Mannan	0.18	0.049	0.10	0.058	0.13
A12	Pectin	0.23	0.071	0.08	0.015	0.10
B1	N-Acetyl-D-Galactosamine	0.22	0.047	0.11	0.063	0.08
B2	N-Acetyl-Neuraminic Acid	0.20	0.068	0.08	0.043	0.08
B3	Beta-D-Allose	0.23	0.085	0.10	0.047	0.11
B4	Amygdalin	0.22	0.079	0.09	0.040	0.10
B5	D-Arabinose	0.38	0.091	0.13	0.060	0.25
B6	D-Arabitol	0.22	0.079	0.13	0.103	0.11
B7	L-Arabitol	0.22	0.074	0.11	0.068	0.12
B8	Arbutin	0.20	0.076	0.11	0.091	0.09
B9	2-Deoxy-D-Ribose	0.73	0.066	0.16	0.108	0.58
B10	i-Erythritol	0.22	0.076	0.10	0.052	0.14
B11	D-Fucose	0.23	0.083	0.10	0.054	0.10
B12	3-0-b-D-Galacto-pyranosyl-D-Arabinose	0.23	0.080	0.14	0.050	0.16
C1	Gentiobiose	0.25	0.053	0.09	0.040	0.12
C2	L-Glucose	0.24	0.081	0.09	0.039	0.11
C3	Lactitol	0.24	0.083	0.10	0.046	0.09
C4	D-Melezitose	0.22	0.078	0.12	0.068	0.15
C5	Maltitol	0.23	0.082	0.10	0.054	0.10
C6	a-Methyl-D-Glucoside	0.26	0.040	0.11	0.066	0.13
C7	b-Methyl-D-Galactoside	0.24	0.079	0.10	0.053	0.09
C8	3-Methyl Glucose	0.23	0.081	0.11	0.065	0.10
C9	b-Methyl-D-Glucuronic Acid	0.21	0.076	0.10	0.055	0.09
C10	a-Methyl-D-Mannoside	0.22	0.087	0.10	0.056	0.10
C11	b-Methyl-D-Xyloside	0.21	0.078	0.11	0.075	0.13
C12	Palatinose	0.23	0.037	0.14	0.044	0.21
D1	D-Raffinose	0.26	0.069	0.07	0.002	0.10
D2	Salicin	0.23	0.074	0.09	0.029	0.09
D3	Sedoheptulosan	0.25	0.080	0.09	0.037	0.09

D4	L-Sorbose	0.27	0.063	0.13	0.078	0.12
D5	Stachyose	0.22	0.074	0.14	0.043	0.09
D6	D-Tagatose	0.33	0.120	0.12	0.061	0.24
D7	Turanose	0.25	0.078	0.13	0.106	0.09
D8	Xylitol	0.24	0.085	0.12	0.049	0.09
D9	N-Acetyl-D-Glucosaminitol	0.22	0.090	0.14	0.069	0.10
D10	g-Amino Butyric Acid	0.23	0.084	0.13	0.087	0.09
D11	d-Amino Valeric Acid	0.20	0.081	0.10	0.040	0.07
D12	Butyric Acid	0.43*	0.158	0.28*	0.144	0.11
E1	Capric Acid	0.25	0.075	0.10	0.027	0.23
E2	Caproic Acid	0.85*	0.049	0.19*	0.134	0.09
E3	Citraconic Acid	0.25	0.055	0.13	0.086	0.10
E4	Citramalic Acid	0.23	0.086	0.12	0.064	0.08
E5	D-Glucosamine	0.66	0.136	0.16	0.102	0.63
E6	2-Hydroxy Benzoic Acid	0.21	0.073	0.10	0.055	0.10
E7	4-Hydroxy Benzoic Acid	0.21	0.075	0.10	0.057	0.10
E8	b-Hydroxy Butyric Acid	0.26	0.085	0.17	0.110	0.09
E9	g-Hydroxy Butyric Acid	0.26	0.098	0.14	0.078	0.11
E10	a-Keto-Valeric Acid	0.27	0.102	0.11	0.070	0.12
E11	Itaconic Acid	0.17	0.076	0.07	0.034	0.06
E12	5-Keto-D-Gluconic Acid	0.62	0.058	0.18	0.081	0.48
F1	D-Lactic Acid Methyl Ester	0.27	0.079	0.12	0.072	0.14
F2	Malonic Acid	0.25	0.087	0.10	0.058	0.10
F3	Melibionic Acid	0.27	0.097	0.09	0.025	0.10
F4	Oxalic Acid	0.24	0.100	0.08	0.038	0.16
F5	Oxalomalic Acid	0.32	0.094	0.12	0.078	0.18
F6	Quinic Acid	0.24	0.084	0.10	0.060	0.11
F7	D-Ribono-1,4-Lactone	0.25	0.076	0.09	0.055	0.07
F8	Sebacic Acid	0.41*	0.104	0.21*	0.120	0.08
F9	Sorbic Acid	0.43	0.133	0.18	0.119	0.31
F10	Succinamic Acid	0.24	0.096	0.12	0.058	0.10
F11	D-Tartaric Acid	0.24	0.079	0.11	0.068	0.09
F12	L-Tartaric Acid	0.24	0.058	0.12	0.063	0.14
G1	Acetamide	0.25	0.066	0.11	0.029	0.09
G2	L-Alaninamide	0.23	0.079	0.12	0.046	0.09
G3	N-Acetyl-L-Glutamic Acid	0.23	0.090	0.12	0.057	0.10

G4	L-Arginine	0.24	0.064	0.12	0.055	0.11
G5	Glycine	0.27	0.100	0.11	0.047	0.09
G6	L-Histidine	0.25	0.097	0.13	0.040	0.14
G7	L-Homoserine	0.25	0.094	0.11	0.061	0.09
G8	Hydroxy-L-Proline	0.25	0.090	0.12	0.059	0.09
G9	L-Isoleucine	0.24	0.094	0.13	0.041	0.08
G10	L-Leucine	0.25	0.095	0.11	0.064	0.12
G11	L-Lysine	0.22	0.068	0.11	0.070	0.08
G12	L-Methionine	0.22	0.053	0.12	0.056	0.11
H1	L-Ornithine	0.23	0.054	0.10	0.040	0.09
H2	L-Phenylalanine	0.24	0.069	0.14	0.065	0.09
H3	L-Pyroglutamic Acid	0.26	0.067	0.12	0.088	0.09
H4	L-Valine	0.24	0.067	0.12	0.049	0.14
H5	D,L-Carnitine	0.22	0.076	0.13	0.066	0.09
H6	Sec-Butylamine	0.23	0.083	0.13	0.058	0.09
H7	D.L-Octopamine	0.25	0.095	0.11	0.069	0.11
H8	Putrescine	0.26	0.062	0.12	0.085	0.18
H9	Dihydroxy Acetone	0.75	0.092	0.18	0.111	0.56
H10	2,3-Butanediol	0.24	0.079	0.10	0.048	0.13
H11	2,3-Butanedione	0.23	0.066	0.08	0.044	0.17
H12	3-Hydroxy 2-Butanone	0.24	0.046	0.08	0.011	0.13

*, considered as a positive phenotype ($p<0.05$ and signal intensity equal or higher than 25%). Unpaired T test was performed. The $O.D._{590nm}$ values are the means of three independent experiments. #, standard deviations (SD) values.
